# Evaluation of fall risk factors for among hospitalized patients: systematic review and meta-analysis

**DOI:** 10.1093/eurpub/ckac131.295

**Published:** 2022-10-25

**Authors:** E Govoni, F La Porta, A Negro, G Valpiani, S Caselli, E Bassi, V Pecoraro, G Lullini, D Tedesco, M Rolli

**Affiliations:** 1 Hospital Care Sector, General Management for People Care, Health, Bologna, Italy; 2 Neurorehabilitation Unit, IRCCS Neurological Sciences Insitute, Bologna, Italy; 3 Regional Agency for Health and Social Care, Emilia Romagna Region, Bologna, Italy; 4 Research and Innovation Office, S. Anna University Hospital, Ferrara, Italy; 5 Rehabilitation Unit, Baggiovara Hospital, AOU Modena, Modena, Italy; 6 Department of Translational Medicine, University of Eastern Piedmont, Novara, Italy; 7 Department of Laboratory Medicine, AUSL Modena, Modena, Italy

## Abstract

**Background:**

Hospitals falls are an important challenge for healthcare systems. An early identification of patients at risk is critical, but no assessing tool has proven to be sufficiently predictive. This review aims at identifying factors associated with an increased risk of falls in hospitalized adults and at mapping them according to main international classification systems.

**Methods:**

We carried out a systematic literature review and metanalysis to detect risk factors positively associated with the increase of falls in hospitals, searching litterature from January 2015 to March 2022. We included studies investigating falling risk factors in patients older than 16 years. Researchers independently assessed records’ eligibility and the methodological quality of included studies was assessed. When possible, data was processed using a random effects model and odds ratio (OR) with 95% confidence interval to quantify the effect. Risk factors were than classified according to ICF, ICD, and ATC classifications.

**Results:**

We included 40 observational studies, enrolling 3,495,552 patients. Considering ICF-factors, mental and sensory functions-pain have a strong association with falls (OR = 3.311 and 2.149, respectively). ICD-factors associated with falls were mental and behavioural disorders (OR = 2.219), diseases of the nervous system (OR = 2.974), and symptoms, signs, and abnormal clinical and laboratory findings (OR = 2.665). Considering ATC-related factors, medications for alimentary tract and metabolism (OR = 1.978), and nervous system (OR = 1.779), showed a strong association with falls. Other factors were also associated with a moderately increased risk.

**Conclusions:**

The comprehensive evidence-based assessment achieved with this meta-analysis alongside with the classification according to ICF, ICD and ATC systems provides a new standardized identification of the risk factors associated with an increase of falls in hospital.

**Key messages:**

• Falls occurring in hospital are an important challenge for health care systems. Therefore the identification of risk factors associated to patients increased risk of fall is fundamental.

• The comprehensive evidence-based assessment achieved with this meta-analysis provides a new standardized identification of the risk factors associated with an increase of falls for hospital.

